# Microcirculation function assessment in acute myocardial infarction: A systematic review of microcirculatory resistance indices

**DOI:** 10.3389/fcvm.2022.1041444

**Published:** 2022-11-11

**Authors:** Marta Silva, Luis Paiva, Rogério Teixeira, Maria João Ferreira, Lino Gonçalves

**Affiliations:** ^1^Faculdade de Medicina, Universidade de Coimbra, Coimbra, Portugal; ^2^Serviço de Cardiologia, Centro Hospitalar e Universitário de Coimbra, Coimbra, Portugal; ^3^Coimbra Institute for Clinical and Biomedical Research, Universidade de Coimbra, Coimbra, Portugal; ^4^Coimbra Institute for Biomedical Imaging and Translational Research, Universidade de Coimbra, Coimbra, Portugal

**Keywords:** myocardial infarction, microvascular dysfunction, microvascular obstruction, hyperemic microvascular resistance, Pzf, microvascular resistance índex

## Abstract

**Background:**

Up to 50% of acute myocardial infarction (MI) patients present with microvascular dysfunction, after a successful percutaneous coronary intervention (PCI), which leads to worse clinical outcomes. The main purpose of this study is to provide a critical appraisal of the emerging role of invasive microvascular resistance indices in the MI setting, using the index of microcirculatory resistance (IMR), hyperemic microvascular resistance (HMR) and zero-flow pressure (Pzf).

**Methods:**

We systematically explored relevant studies in the context of MI that correlated microcirculation resistance indices with microvascular dysfunction on cardiac magnetic resonance (CMR), microvascular dysfunction occurring in infarct related arteries (IRA) and non-IRA and its relation to clinical outcomes.

**Results:**

The microcirculation resistance indices correlated significantly with microvascular obstruction (MVO) and infarct size (IS) on CMR. Although HMR and Pzf seem to have better diagnostic accuracy for MVO and IS, IMR has more validation data. Although, both IMR and HMR were independent predictors of adverse cardiovascular events, HMR has no validated cut-off value and data is limited to small observational studies. The presence of microvascular dysfunction in non-IRA does not impact prognosis.

**Conclusion:**

Microvascular resistance indices are valuable means to evaluate microcirculation function following MI. Microvascular dysfunction relates to the extent of myocardial damage and clinical outcomes after MI.

**Systematic review registration:**

[https://www.crd.york.ac.uk/prospero/display_record.php?ID=CRD42021228432], identifier [CRD42021228432].

## Introduction

Many patients following an acute myocardial infarction (MI) have adverse clinical outcomes despite a successful percutaneous coronary intervention (PCI) (1). Solely revascularizing an obstructed epicardial coronary artery may not be enough to improve microcirculation in acute coronary syndromes (ACS), and persistent microvascular dysfunction leads to worse clinical outcomes (2).

**FIGURE 1:**
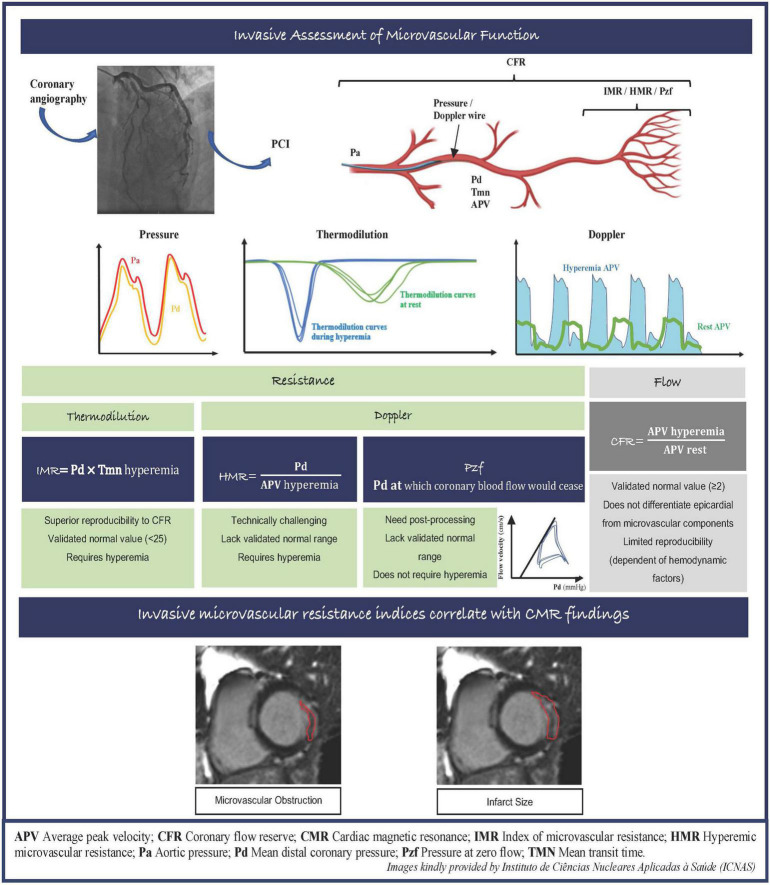
Invasive assessment of microvascular function and correlation between invasive microvascular resistance indexes with CMR images in the MI setting. APV, average peak velocity; CFR, coronary flow reserve; CMR, cardiac magnetic resonance; IMR, index of microvascular resistance; HMR, hyperemic microvascular resistance; Pa, aortic pressure; Pd, mean distal coronary pressure; Pzf, pressure at zero flow; TMN, mean transit time.

Several vascular resistance indices have been proposed to evaluate microcirculation function invasively, that have the advantage of being available in the catheterization laboratory to delineate the specific contribution of microcirculation to myocardial damage. The index of microvascular resistance (IMR) is a thermodilution technique that allows the quantitative assessment of the microcirculatory resistance in a coronary artery territory. The hyperemic microvascular resistance index (HMR) represents the ratio of mean distal coronary pressure (Pd) and doppler flow peak velocity during hyperemia. Lastly, zero-flow pressure (Pzf) that is extrapolated from pressure-velocity plots, represents Pd at which the coronary blood flow would cease. As opposed to coronary flow reserve (CFR), vascular resistance indices are specific for the microcirculation and independent of hemodynamic variations, making IMR more reproducible than CFR (3). The key characteristics of IMR, HMR, and Pzf are summarized in the Figure 1.

Microvascular obstruction (MVO) is an entity that represents the inability to reperfuse the coronary microcirculation within the infarct zone. It has been associated with major adverse clinical outcomes and it is considered a stronger prognostic marker than infarct size (IS) (3, 4). MVO can be assessed through different procedures at different timings after MI, however, contrast-enhanced cardiac magnetic resonance (CMR) is the gold standard technique (5).

The main purpose of this study is to provide a critical appraisal of the emerging role of microvascular resistance indices in the MI setting, its relation to MVO and IS on CMR, and its relation to clinical outcomes in the infarct related arteries (IRA) and non-IRA.

**FIGURE 2:**
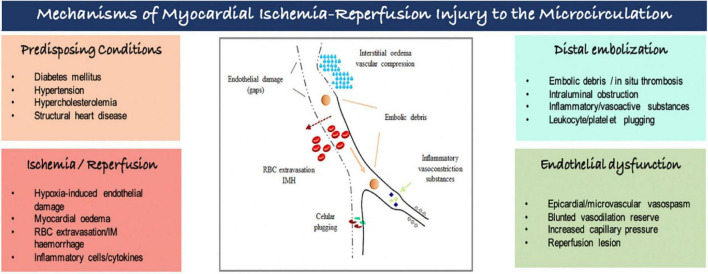
Mechanisms of myocardial ischemia-reperfusion injury to the microcirculation. IM, intramyocardial; RBC, red blood cell.

Several and interconnected pathomechanisms had been proposed to explain myocardial ischemia-reperfusion injury to the microcirculation (Figure 2). There is increasing evidence that ischemia affects not only cardiomyocytes, but also endothelial cells, despite its greater resistance to hypoxemia. Endothelial dysfunction is characterized by (i) endothelial protrusions and blebs, which lead to capillaries obstruction; (ii) endothelial gaps, allowing red blood cells leakage into the myocardial *interstitium*, causing intramyocardial hemorrhage; and extravasation of fluids, leading to interstitial edema and greater compression of the vessel’s lumen. The capillary dysfunction is further amplified by a proinflammatory and proaggregant environment caused by increased concentrations of vasoconstrictors substances (i.e., thromboxane A2, endothelin), and by the expression of adhesion molecules in vessels and circulating cells promoting the formation of neutrophil-platelet and erythrocytes aggregates causing obstruction of the capillaries. Furthermore, distal embolization originated from micro emboli detached of coronary plaques and/or released during PCI can worsen microcirculatory ischemic injury. Individual’s susceptibility determined by some genetic polymorphisms, pre-existing cardiovascular comorbidities and advanced age augments the risk of coronary microvascular dysfunction associated with MI (5–7).

Cardioprotection refers to interventions that reduce the injury from myocardial ischemia and reperfusion. Mechanical ischemic conditioning approaches, involving brief cycles of ischemia-reperfusion (e.g., arm cuff inflation-deflation cycles) reduce IS and coronary microvascular damage, through a complex signal transduction pathway that target intracellular organelles such as mitochondria and sarcoplasm reticulum (8). Pharmacological cardioprotection largely aimed at either inhibition of injurious pathways or activation of protective processes, such as increased formation of adenosine or nitric oxide. However, most published data did not show a significant impact of adenosine or nitrite therapy on MVO or in clinical outcomes (8, 9). Similarly, several other therapies used early in the acute MI setting (e.g., P2Y12 inhibitors, glycoprotein IIb/IIIa inhibitors, fibrinolytics) that induced cardioprotection in preclinical models, often failed to confirm its efficacy in clinical trials. A promising approach would be the additive cardioprotection, through proven mechanical (combined ischemic pre and postconditioning) and pharmacological strategies, in addition to reperfusion, and focusing on MI patients that would benefit the most from cardioprotection measures, such as those in Killip class III or cardiogenic shock (8).

## Materials and methods

### Protocol and registration

This systematic review was performed in accordance with Preferred Reporting Items for Systematic Reviews and Meta-Analysis (PRISMA) standard and is registered in PROSPERO database (CRD42021228432).

### Information sources

**FIGURE 3:**
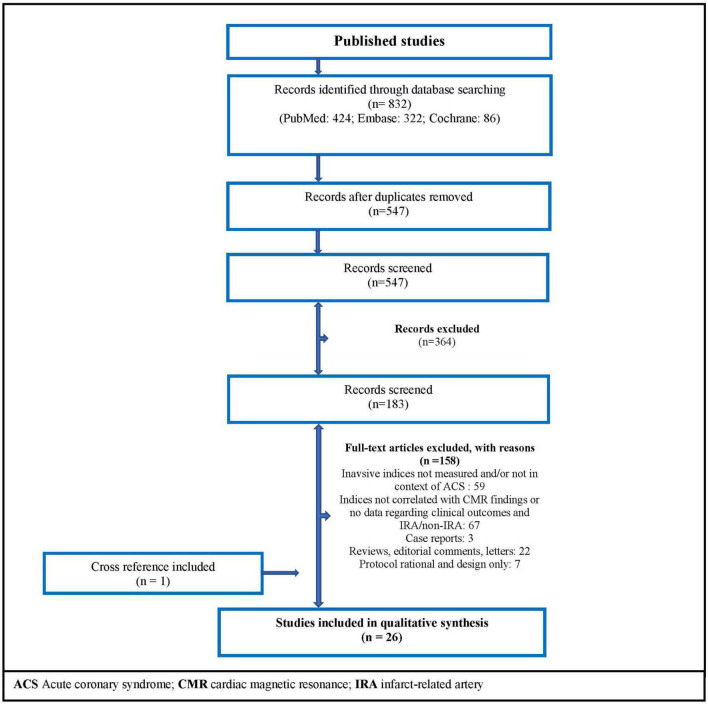
Flow diagram of the search strategy. ACS, acute coronary syndrome; CMR, cardiac magnetic resonance; IRA, infarct-related artery.

A systematic search was performed on PubMed, Embase, and Cochrane Controlled Register of Trials on June 17, 2021. The search terms are presented in the Supplementary Table 1. Interventional and observational original studies were included, and there was no date restriction. Figure 3 shows PRISMA flow diagram related to our search strategy.

### Eligibility criteria

The eligible studies reported associations between acute MI patients and microvascular resistance indices (IMR, HMR, and Pzf) – measured invasively in a cardiac catheterization laboratory – and CMR findings of microcirculation dysfunction, using MVO and cardiac viability. Furthermore, the selected studies included data on clinical outcomes of the infarct related arteries and non-IRA.

In order to perform a random-effect meta-analysis in patients with and without MVO by CMR, only studies reporting mean values ± standard deviation (SD) of the microvascular resistance indices (IMR, HMR, and Pzf) were included.

### Data collection process

One author (M. Silva) systematically screened the titles and abstracts of publications retrieved using the search strategy mentioned above to verify inclusion criteria. The full texts of the selected studies were, again, independently reviewed for eligibility by two co-authors. For the performance of the random-effect meta-analysis, only four studies reported mean values ± SD of microvascular resistance indices and two co-authors performed data extractions independently (10–13).

### Quality assessment

Two investigators (M. Silva/L. Paiva) assessed the risk of bias of the included studies, following the Newcastle-Ottawa Scale for observational studies (Supplementary Table 2). None of the non-randomized studies demonstrated that the outcome of interest was not present at the beginning of the study. Furthermore, most included studies did not have a control group, reducing the comparative capacity and underpowering the conclusions reached. Despite this, the assessment of outcomes, follow-up time and adequacy were accurate in most studies.

### Statistical analysis

RevMan 5.4 (Nordic Cochrane Centre) was used to conduct a random-effect meta-analysis, with the effect measured by standardized mean difference (for collected IMR, HMR, and Pzf data) and mean difference (for only IMR studies) with 99% confidence intervals. All reported p values are two-sided, with significance set at *P* < 0.05. Heterogeneity among trials was quantified using *I*^2^ statistics.

## Results

### Search results

Twenty-six studies met the inclusion criteria (Figure 3), with no randomized controlled trial included. Subdividing articles by topics: 14 non-randomized studies related to CMR findings; 4 observational articles showed data about direct correlations between resistance indices; 9 studies reported clinical outcomes; and 8 observational papers described non-culprit artery microvascular findings after MI.

### Vascular resistance indices and microvascular obstruction/infarct size by cardiac magnetic resonance

Several articles explored the association between MVO and IS quantified by CMR, and invasive resistance indices, mainly using IMR measurements. The timing of the CMR after MI varied between studies (Table 1). Most studies (80%) reported the cardiac imaging in <7 days after the myocardial ischemic event, ranging between 1 day and 2 weeks following the ischemic event and some studies (50%) had an additional CMR study 3–6 months after the index event. Furthermore, IMR is a functional measure of the microvascular viability continuum within the distribution of a coronary artery. Patients without microvascular disease typically present IMR values < 25, while higher values of IMR correlate with impaired myocardium perfusion (14).

**TABLE 1:** Non-randomized controlled trials of microvascular resistance indices in acute MI associated with MVO and IS on CMR.

CMR				Cardiac imaging associations with microcirculation measures			Comparation between indices
		
First author/Study	Sample size	Index	Timing after MI	IMR	HMR	Pzf	
Williams et al. (1)	**44** Acute MI	**IMR, HMR** after PPCI	1 day	IMR (*r* = 0.36; *p* = 0.01) correlated with MVO	HMR (*r* = 0.46; *p* = 0.001) correlated with MVO	—————————–	IMR correlated with HMR (*r* = 0.39; *p* = 0.0006) HMR was not significantly superior over IMR to predict MVO (AUC 0.75 vs. 0.66)
Patel et al./OXAMI study (2)	**34** STEMI	**IMR, HMR, Pzf** after PPCI	6 months	IMR did not correlate with MVO and IS Not a predictor of ≥ 24% of infarction (AUCIMR 0.54; *p* = 0.77)	HMR correlated to IS (*r* = 0.54, *p* = 0.009) Predictor of ≥ 24% of infarction: AUCHMR 0.74 (*p* = 0.04)	Pzf correlated to MVO (*r* = 0.49; *p* = 0.02) and IS (*r* = 0.77; *p* < 0.0001) Predictor of ≥ 24% of infarction: AUCPzf 0.94 (*p* < 0.0001). Optimal cut-off was 42 mmHg (100% sensitivity, 73% specificity)	Pzf was a better predictor of IS than HMR (*p* = 0.04) or IMR (*p* = 0.03)
DeMaria et al. (14)	**110** STEMI	**IMR** after PPCI	2 days and 6 months	IMR correlated with MVO (*r* = 0.29, *p* = 0.002) and IS at 48 h (*r* = 0.21, *p* = 0.03) and 6 months (*r* = 0.43, *p* = 0.001)	————————	————————–	————————
McAlindon et al./MICRO-AMI study (15)	**50** STEMI	**IMR** after PPCI	2–4 days and 3 months	IMR correlated (*r* = 0.61; *p* < 0.001) and was predictive of MVO (AUC 0.78), optimal cut-off was 40 (sensitivity 59%, specificity 92%) IMR not associated with IS	————————	————————	————————-
Teunissen et al. (10)	**60** STEMI	**HMR, Pzf** after PPCI	4–6 days	———————————————-	HMR correlated with MVO (*r* = 0.46; *p* < 0.01) and IS (*r* = 0.41; *p* < 0.01) Predictor of MVO: AUC_*HMR*_ 0.68 (*p* = 0.03)	Predictor of MVO: AUC_*Pzf*_ 0.75 (*p* = 0.01)	HMR and Pzf were Significantly correlated (*r* = 0.55; *p* = 0.002)
Yoo et al. (11)	**34** Acute MI	**IMR** after PPCI	6 ± 4 days	IMR correlated with MVO (*r* = 0.75; *p* < 0.001)	————————	————————	————————
Ahn et al. (12)	**40** STEMI	**IMR** after PPCI	7 days	IMR associated (OR 1.15, 95% CI 1.05–1.26) and was predictive of MVO (AUC 0.87; *p* < 0.001); optimal cut-off was 27U (74% sensitivity, 88% specificity)	————————	————————	————————
Kitabata et al. (16)	**27** Acute MI	**HMR, Pzf** after PPCI	13 ± 2 days	———————————————-	HMR was significantly correlated to IS (*r* = 0.78, *p* < 0.0001)	Pzf was significantly correlated to IS (*r* = 0.72, *p* = 0.0002)	HMR correlated with Pzf (*r* = 0.75, *p* < 0.0001)
Maznyczka et al. (17)	**144** STEMI	**IMR** after PPCI	2–7 days and 3 months	IMR correlated to MVO (*r* = 0.20; *p* = 0.016) at 2–7 days IMR and IMR > 40U were associated with IS at 3 months (OR 0.12; *p* < 0.001 and OR 9.12; *p* < 0.001, respectively)	————————	————————	————————
Carrick et al. (19)	**283** STEMI	**IMR** after PPCI	2 days and 6 months	IMR > 40U was independently associated with MVO, 2 days after MI (OR 2.82; *p* < 0.001)	————————	————————	————————
DeWaard et al. (20)	**176** Acute MI	**HMR** after PPCI	24 h to 2 weeks	———————————————	HMR significantly predicted MVO (AUC of 0.76; 95% CI 0.67–0.85)	————————	————————
Scarsini et al./Insights from OXAMI study (35)	**45** STEMI	**IMR** after PPCI	2 days and 6 months	IMR predicted IS at 48 h (AUC = 0.71; 95%CI 0.71–0.99) and was significantly correlated to IS at 6 months (*r* = 0.35, *p* = 0.027)	————————	————————	————————
Scarsini et al.(44)	**148** STEMI	**IMR** after PPCI	2 days and 6 months	IMR predicted MVO at 48 h (OR 1.02; *p* = 0.008) and IS at 48 h (OR 1.01; *p* = 0.022) and 6 months (OR 1.02; *p* = 0.017)	————————	————————	————————
Maznyczka et al. (45)	**271** STEMI	**IMR** after PPCI	2–7 days	IMR_*AUC*_ of 0.69 (*p* < 0.001) for predicting presence of MVO	————————	————————	————————

AUC, area under the curve; CI, confidence interval; CMR, cardiovascular magnetic resonance; HMR, hyperemic microvascular resistance; IMR, index of microvascular resistance; IS, infarct size; MI, myocardial infarction; MVO, microvascular obstruction; PPCI, primary percutaneous coronary intervention; Pzf, zero-flow pressure; STEMI, ST segment elevation myocardial infarction.

Regarding MVO, de Maria et al. (14) reported a significant but weak correlation with IMR as a continuous variable (*r* = 0.29). When IMR was analyzed with a cut-off of > 40 (higher specificity for microvascular dysfunction), more than one-third of STEMI patients presenting MVO had IMR values ≤ 40. Nonetheless, most of these discordant cases had IMR values between 25 and 40, and at 6 months of follow-up, those patients had a significant regression in the myocardium scar size, while patients presenting with both MVO and IMR > 40 had no scar regression. Despite this, in a previous study MICRO-AMI, IMR presented a stronger relationship to MVO (*r* = 0.61, *p* < 0.001) after MI, especially in those with IMR > 40 (15).

The microvascular resistance indices using doppler flow velocity (HMR and Pzf) showed a significant correlation with MVO, however, both indices lack validated normal range values. Williams et al. (1) reported that both IMR and HMR measurements correlated with MVO (IMR: *r* = 0.36, *p* = 0.01; HMR: *r* = 0.46, *p* = 0.001), however, HMR accuracy to predict MVO was not significantly superior to IMR (HMR_*AUC*_ 0.75 vs. IMR_*AUC*_ 0.66). Importantly, the OxAMI study (2) analyzed all three indices and reported a significant correlation between Pzf and MVO (*r* = 0.49, *p* = 0.02), which was a significantly better predictor of infarct extension than HMR or IMR. Regarding their diagnostic accuracy for MVO, ROC curve analysis showed non-significant differences, in decreasing order of size: Pzf, HMR, and IMR.

**FIGURE 4:**
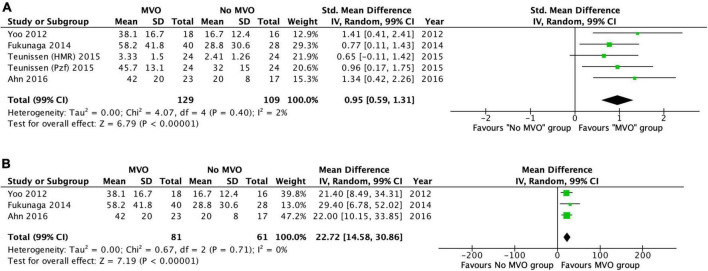
**(A)** Forest plot of IMR, HMR, and Pzf in patients with and without MVO by CMR. Forest plot of the six studies included and shows the weighted standardized mean difference of vascular resistance indices in those with and without microvascular obstruction (MVO) by cardiac magnetic resonance imaging (CMR). **(B)** Forest plot of IMR in patients with and without MVO by CMR. Forest plot of the four index microvascular resistance (IMR) studies included and shows the weighted mean difference of IMR in those with and without microvascular obstruction (MVO) by cardiac magnetic resonance imaging (CMR).

We conducted a meta-analysis in four of the included studies (10–13), which reported mean values ± SD of IMR, HMR and Pzf in patients with and without MVO by CMR. It comprised a total of 238 cases and further details of these studies are presented in Table 1. MVO by CMR was present in 49% of the patients. The standardized weighted mean difference in the three vascular resistance indices between patients with and without MVO was 0.95 (99% confidence interval [CI]: 0.59–1.31; *I*^2^ = 2%; *p* < 0.00001) (Figure 4A). Subsequently, we perform a random-effect meta-analysis that included only IMR data (three of the four included studies), comprising 142 patients (11–13). The weighted mean difference in IMR between the two groups was 22.7 (99% CI: 14.6–30.9; *I*^2^ = 0%; *p* < 0.00001) (Figure 4B). The weighted mean IMR in the 81 patients with MVO was 49.1 (99% CI: 46.4–51.8), whereas it was 23.6 (99% CI: 21.8–25.4, *p* < 0.0001) in the 61 patients without MVO.

Concerning IS measured by CMR, IMR showed no or weak correlation in the early days after STEMI (<4 days) (14, 15). However, in delayed CMR, the relationship between indices and IS becomes stronger. Teunissen et al. (10) reported a low to moderate correlation with IS (HMR: *r* = 0.41, *p* < 0.001) if CMR measures were obtained 4–6 days after STEMI and a stronger correlation was found (HMR: *r* = 0.78; *p* < 0.001; Pzf: *r* = 0.72, *p* < 0.001) if CMR was attained more than 10 days after MI (16). At 6 months follow-up, the OxAMI study reported a significant correlation between IS and HMR (*r* = 0.54, *p* = 0.009) and with Pzf (*r* = 0.77, *p* < 0.001) (2). Other studies confirmed that correlations between invasive indices and IS are stronger > 10 days after MI, which relates to scar formation and cardiac remodeling timing (14).

### Microvascular resistance indexes and clinical outcomes

Microvascular dysfunction is increasingly being recognized as an important marker of adverse clinical events in MI patients (Table 2).

**TABLE 2:** Non-randomized controlled trials of microvascular resistance indices in acute MI associated with clinical outcomes.

First author/Study	Sample size	Index	Follow-up time	Association with clinical outcomes
Scarsini et al. (4)	**198** STEMI	**IMR** after PPCI	Median 40.1 months	At 1 year and at long term follow-up, group with IMR > 40 and MVO had a significantly higher risk of adverse clinical outcomes than group with IMR ≤ 40 and no MVO (HR 12.6; *p* = 0.017 and HR 5.2; *p* = 0.004). At 1 year follow-up, group with IMR > 40 or MVO showed no significant differences compared to group with IMR ≤ 40 and no MVO, however, at long term follow-up, adverse clinical outcomes were higher in the first group (HR 4.2; *p* = 0.009) and similar to those with IMR > 40 and MVO
Fukunaga et al. (13)	**88** STEMI	**IMR** after PPCI	6 months	Optimal cut-off of IMR for cardiac death, non-fatal MI and HHF was 37U (AUC 0.68, sensitivity 75%, specificity 61%). IMR > 37 was associated with a higher incidence of MACE versus IMR ≤ 37 (*p* = 0.007). IMR was not an independent predictor of MACE (HR 0.99; *p* = 0.59)
Maznyczka et al. (17)	**144** STEMI	**IMR** after PPCI	1 year	IMR, IMR > 40U and IMR > 44U were associated with HHF (OR 1.02, *p* < 0.001; OR 5.34, *p* = 0.002; OR 6.92; *p* = 0.001, respectively) IMR (OR 1.02, *p* = 0.001), IMR > 40U (OR 4.08, *p* = 0.005) and IMR > 44U (OR 5.33, *p* = 0.001) were also associated with death and HHF
Fearon et al. (18)	**253** STEMI	**IMR** after PPCI	Median 2.8 years	IMR > 40U was a predictor of death (HR 4.30; *p* = 0.02) and death or HHF (HR 2.20; *p* = 0.03)
Carrick et al. (19)	**283** STEMI	**IMR** after PPCI	Median 845 days	IMR > 40U associated with all-cause death or HHF (OR 4.36; *p* < 0.001)
De Waard et al. (20)	**176** acute MI	**IMR** after PPCI	Median 3.2 years	HMR > 3.0 mmHgcm–1s was associated with death (HR 6.4, 95%CI: 1.3–32.0) and HHF (HR 7.0; 95%CI: 1.5–33.7)
Jin et al. (21)	**145** STEMI	**IMR** after PPCI	Mean 85 ± 43 months	HMR optimal cut-off of 2.82 mmHgcm–1s (AUC 0.82; p = 0.006) predicted cardiac death and HHF HMR > 2.82 mmHgcm–1s was associated with MACE (HR 1.74; *p* < 0.001)
Maznyczka et al. (45)	**271** STEMI	**IMR** after PPCI	5 years	IMR was a predictor of death or HHF and MACE at 30 days (AUC_*IMR*_ 0.74; *p* < 0.001 and AUC_*IMR*_ 0.74, *p* = 0.002, respectively) and 5 years after MI (AUC_*IMR*_ 0.64, *p* = 0.002 and AUC_*IMR*_ 0.66, *p* < 0.001, respectively)
Fahrni et al./Insights from OxAMI cohort (46)	**260** STEMI	**IMR** after PPCI	30 days	IMR was a predictor of MACE (AUC 0.90; 95% CI 0.85–0.93); IMR ≤ 40U identified all patients free of major cardiac events (100% sensitivity, 62% specificity)

AUC, area under the curve; CI, confidence interval; HHF, hospitalization for heart failure; HMR, hyperemic microvascular resistance; IMR, index of microvascular resistance; MACE, major adverse cardiac events; MI, myocardial infarction; PPCI, primary percutaneous coronary intervention; STEMI, ST segment elevation myocardial infarction.

In a subset of 144 STEMI patients from the T-TIME trial (17), IMR > 40 was associated with heart failure (HF) hospitalization (OR 5.34, *p* = 0.002) and all-cause death/HF hospitalization (OR 4.08, *p* = 0.005), predicting higher risk of MACE at 1-year of clinical follow up. Fearon et al. (18) reported that acute MI patients with IMR > 40 had a two times higher relative risk of hospitalization for HF (*p* = 0.034) and four times greater risk of all-cause death (*p* = 0.028), and IMR > 40 was the only independent predictor of death. Furthermore, Carrick et al. (19) studied 283 STEMI patients and categorized them accordingly to IMR (≤40 or >40) and coronary flow reserve (CFR) (≤2.0 or >2.0), measured at the end of PCI. They concluded that IMR had superior prognostic value for risk stratification of death or HF, then other traditional markers of myocardial reperfusion, such as symptom-to-reperfusion time, angiographic blush grade or CFR.

A recent study by de Waard et al. (20) using HMR to measure microvascular dysfunction after MI, reported that HMR can be used to detect patients at risk of adverse clinical outcomes. A cut-off value of 3.0 mmHgcm^–1^, was a significant predictor of death and hospitalization for HF, superior to CFR predictive value. These findings were corroborated by Jin et al. (21), showing HMR as an independent predictor of future cardiovascular events. The prognostic ability of Pzf was not yet assessed in clinical studies.

More recently, an important study by Scarsini et al. (4) evaluated the long-term (median follow up of 40 months) prognostic implications of microvascular dysfunction, measured as MVO on CMR and invasively using the cut-off value of IMR > 40, in 198 STEMI cases. Patients were classified as having no significant microvascular dysfunction (group 1: IMR ≤ 40 and no MVO), microvascular dysfunction with either high IMR (>40) or MVO (group 2), and patients with both IMR > 40 and MVO (group 3). At 1-year follow up, group 3 (HR 12.6, *p* = 0.017) but not group 2 had worse clinical outcomes compared with those patients without microvascular dysfunction in group 1. However, in the long-term, group 2 (HR 4.2, *p* = 0.009) and group 3 (HR 5.2, *p* = 0.004) showed similar adverse outcomes, mainly driven by HF. The authors concluded that microvascular dysfunction defined either invasively (IMR > 40) or by CMR-assessed MVO, exhibited a similar risk for adverse outcomes in the follow up.

### Microcirculation in non-culprit infarct related arteries

More than 50% of STEMI patients present with multivessel coronary disease and PCI of non-culprit vessels among these patients is associated with improved clinical outcomes compared to culprit vessel–only PCI (22).

Data on non-culprit IRA using microvascular resistance indices is limited and based in non-randomized trials with small sample sizes (Table 3). Choi et al. (23) enrolled 100 MI cases that underwent a comprehensive coronary physiologic assessment after primary PCI and compared them to patients with stable ischemic heart disease (SIHD). Microcirculation function was evaluated in culprit and non-culprit IRA of both acute MI and SIHD patients. The authors reported that IMR was significantly higher in the culprit IRA rather than non-culprit arteries (33.0 ± 21.0 vs. 17.9 ± 10.5; *p* < 0.001). However, IMR was not different between non-culprit vessel of acute MI and SIHD patients (18.5 ± 11.4). Later on, Mejía-Rentería et al. (24), also using IMR, and Teunissen et al. (10), through HMR, reached the same conclusion: microvascular damage is predominantly localized in the culprit IRA in STEMI patients.

**TABLE 3:** Non-randomized controlled trials of microvascular resistance indices in acute MI measured in non- infarcted arteries.

First author/Study	Sample size	Index	IRA/Non-IRA	IRA and non-IRA microcirculation measures
Teunissen et al. (10)	**60** STEMI	**HMR** after PPCI	IRA and non-IRA	HMR in IRA was higher vs. control (2.87 ± 1.45 vs. 2.26 ± 0.83 mmHgcm–1s; *p* = 0.02). HMR in non-IRA vs. control no different. A significant increasing HMR trend was found between control < non-IRA < IRA (*p* < 0.01). HMR was higher in patients with vs. without MVO only in IRA (3.33 ± 1.50 vs. 2.41 ± 1.26 mmHgcm–1s; *p* = 0.03)
Ntalianis et al. (22)	**14** acute MI	**IMR** after PPCI and 3 months later	IRA and non-IRA	IMR values on non-IRA lesions were found in normal range (<30U) in 79% of patients. These values did not change during follow-up (4 days to 3 months later)
Choi et al. (23)	**100** acute MI	**IMR** after PPCI	IRA and non-IRA	IMR was higher in IRA than non-IRA (33.0 vs. 17.9U, *p* < 0.001); and control (vs. 18.5U, *p* < 0.001); IMR was not significantly different in non-IRA vs. control.
Mejía-Rentería et al. (24)	**49** acute MI	**IMR** 6 days after MI	Non-IRA	IMR in non-IRA vs. control was not significantly different (15.6 non-IRA vs. 16.7U control) in the subacute phase of MI
Díez-Delhoyo et al./FISIOIAM study (25)	**84** STEMI	**IMR** after PPCI	Non-IRA	IMR > 25U in non-culprit lesions in 28% of cases. Macrovascular and microvascular dysfunction were not correlated with each other
Van der Hoeven et al. /REDUCE-MVI substudy (28)	**73** STEMI	**IMR** after PPCI and 1 month	Non-IRA	IMR decreased from index event to 1-month (18.0 vs. 14.5U, *p* = 0.06). IMR correlated to myocardial salvage index (*r* = –0.43; *p* = 0.001)
Bax et al. (29)	**73** acute MI	**HMR** after PPCI	IRA and non-IRA	HMR of IRA was higher vs. non-IRA at the acute event (3.2 ± 1.7 vs. 2.2 ± 1.7 mmHgcm–1s). HMR in IRA showed a significant decreased from acute to 1 week and 6 months follow-up (3.2 > 2.0 > 1.8 mmHgcm–1s). In non-IRA, HMR decreased from acute to 1 week, but stabilized at 6 months (2.2 > 1.7, *p* < 0.0001 and 1.8 mmHgcm–1s, respectively)
De Maria et al. (47)	**45** STEMI	**IMR** before and after PPCI	Non-IRA	15 STEMI had non-IRA measures: IMR was higher in IRA vs. non-IRA (31 vs. 19U; *p* = 0.01)

HMR, hyperemic microvascular resistance; IMR, index of microvascular resistance; IRA, infarct related artery; MI, myocardial infarction; PPCI, primary percutaneous coronary intervention; STEMI ST, segment elevation myocardial infarction.

Despite this, some studies (22, 25) found that IMR in the non-culprit arteries was abnormally high (IMR > 25) in 21–28% of the acute MI patients and other trials reported that MI patients had a depressed myocardial stress perfusion, particularly in the infarcted but also in non-infarcted regions (26, 27). The REDUCE-MVI substudy (28) selected 98 STEMI patients that had performed microvascular function assessment of intermediate coronary lesions at least in one non-culprit IRA at primary PCI and 1 month later. The authors found a blunted vasodilatory response of the microcirculation to adenosine at the acute event, which was more pronounced in patients with large IS, low left ventricular ejection fraction (LVEF) and higher microvascular injury. The IMR values in non-IRA decreased in the follow-up (from 18.0, [IQR 13.5–27.0] to 14.5 [IQR 11.0–21.0], *p* = 0.06), and both IMR in the acute setting and IMR temporal changes in non-IRA correlated significantly with myocardial salvage index on CMR (29).

## Discussion

**FIGURE 5:**
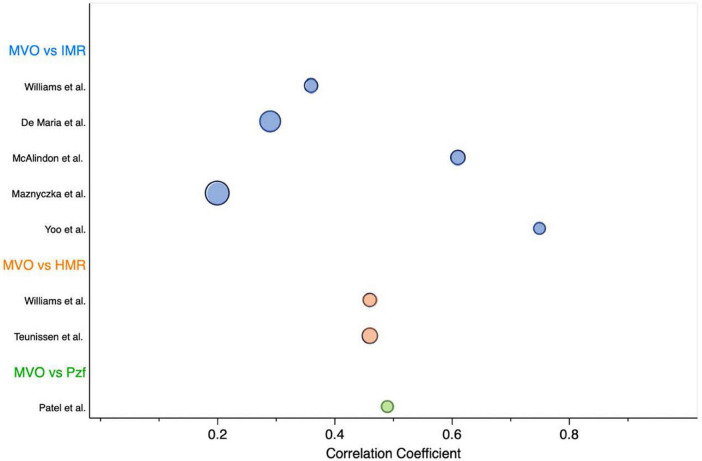
Overview of studies evaluating the correlation coefficient between MVO by CMR and invasive microvascular resistance indices. Bubble chart sized according to the sample size of each study. HMR, hyperemic microvascular resistance; IMR, index of microvascular resistance; MVO, microvascular obstruction; Pzf, pressure at zero flow.

We provided an overview of currently used resistance invasive techniques to assess coronary microvascular function following an acute MI and examined its relationship with MVO/IS by CMR and its relation to clinical outcomes regarding the culprit and non-culprit IRA. We found substantial differences between reported invasive measurements of microvascular dysfunction and MVO by CMR, which are summarized in Figure 5. These differences reflect the heterogeneity found in the study’s protocols (e.g., type of invasive technique, timing between invasive/non-invasive measurements), which limit the performance of formal meta-analysis or give clinical meaningful cut-off values for the doppler resistance indices (HMR and Pzf). Although, there is no true reference standard for invasive measurement of microvascular function, the vast majority of data derives from IMR and, importantly, HMR and Pzf lack a well-validated normal range.

Previously, several diagnostic tools have been proposed to identify microvascular injury in the catheterization laboratory. Angiographic parameters like the amount of time required contrast dye to reach a distal segment of a coronary artery (corrected TIMI frame count), clearing of myocardial blush after contrast injection (TIMI myocardial perfusion grade, TMPG), or according to myocardial blush intensity (myocardial blush grade, MBG). However, these angiographic methods are not quantitative and its visual assessment has limited reproducibility and does not make possible a real-time evaluation of microvascular function (post-processing). Studies have found that TMPG is the most valuable of these angiographic techniques in assessing MVO and IS (30, 31). Regarding MBG it is correlated with myocardial reperfusion after PCI, and some studies proved that it can also be predictive of MVO (12). However, it is a semiquantitative measure, with a considerable intra and interobserver variability, and presents a weak to moderate correlation strength to IMR (*r* = –0.42; *p* = 0.007) (32). Furthermore, Carrick et al. (33) reported that MBG has inferior clinical value for risk stratification than IMR, and data comparing it to HMR and Pzf is lacking.

As a continuous variable, IMR correlation with MVO and IS largely varied, ranging from no (2) to weak (1, 14, 17), moderate (15) or strong strength of relationship (11). These inconsistent results between IMR and the presence of MVO may be likely due to being underpowered studies. Bulluck et al. (34) conducted a meta-analysis to evaluate the role of IMR in detecting MVO at the time of primary PCI in STEMI patients. This study suggested that patients with a mean IMR > 41 were more likely to present MVO. We provided a meta-analysis using resistance measurements of IMR, HMR and Pzf. Our results suggested that MI patients with higher standardized weighted mean values favored the identification of MVO in CMR. The overall effect size was highly significant and with a very low variation across studies. Subsequently, we conducted a meta-analysis using only IMR studies, comprising a small number of cases (*N* = 142). Nonetheless, our results show that a weighted mean IMR of < 25 (upper limit of the 99% CI in group without MVO) were less likely to have MVO, while those with a weighted mean IMR of > 46 (lower limit of the 99% CI in the group with MVO) were likely to have MVO by CMR. Interestingly, an IMR cut-off value of > 40 had already been validated in previous studies to identify MVO and MACE in the MI setting. Also, the IMR cut-off < 25 is typically used to define patients without microvascular dysfunction in the literature.

Regarding doppler-derived indices (HMR, Pzf), they seemed to relate more consistently with CMR findings than IMR (1, 2), however, no significant differences were found in their diagnostic accuracy to identify MVO. Overall, the resistance indices presented a modest effect size correlation between them: IMR vs. HMR (*r* = 0.39, *p* < 0.001), HMR vs. Pzf (*r* = 0.55, *p* = 0.002; *r* = 0.75, *p* < 0.001) (1, 10, 16), which suggest that the three resistance indices may not be considered equivalent techniques for measuring microvascular function in the MI setting. Furthermore, IMR is known to be a reproducible approach to measure microvascular resistance in the acute setting. HMR is a more challenging technique due to specific doppler-flow-velocity tracing methodological procedures, leading to a more operator-dependent procedure, with a higher likelihood of a wider range of values, making it more difficult to determine a valid normal range of HMR values (see Figure 1) (10).

Novel microcirculatory indices are emerging, such as the Resistive Reserve Ratio (RRR). An intracoronary thermodilution parameter, which derives from IMR (ratio of basal resistance to IMR). Whereas IMR measures at peak hyperemia and reflects microcirculatory structure, RRR quantifies the vasodilator response of coronary microcirculation to a hyperemic stimulus. By expressing vascular resistance during resting conditions and maximal hyperemia, it can be interpreted as the ability of microcirculation to recover after an acute ischemic injury. Scarsini et al. reported that RRR may offer incremental prognostic value compared to other thermodilution-derived indices, such as IMR and CFR, in predicting the extent of MI in patients with STEMI (35). However, the limited sample size (*N* = 45) and retrospective design of the study, the lack of a validated threshold for RRR, overall limits the reproducibility of their results. Nevertheless, it has been observed that impaired microvascular vasodilatory function occurs in the presence of prolonged ischemia and MVO (36) and can predict HF hospitalizations (17). Larger studies are needed to define whether RRR adds significant information to the IMR in assessing microvascular function after MI.

Often studies that use myocardial hyperemia, do not consider patients with a blunted systemic vasodilation and reflex tachycardia (heart rise < 10 bpm) after an adenosine infusion. Patients with heart failure, diabetes and polymorphisms of adenosine A2a receptors, may present this reduced hemodynamic response to adenosine, but it is uncertain to what extent this lack of peripheral response is reflected in coronary vasodilation. Previously, Mishra et al. have reported that despite the lack of peripheral response to adenosine seen in some patients, coronary vasodilatation remained adequate for the purpose of myocardial perfusion imaging (37). A recent study compared stress myocardial blood flow (MBF) and hemodynamic response with different dosing regimens of adenosine during stress perfusion CMR (38). The authors reported that, although non-responders to standard dose adenosine had a significantly higher stress heart rate following a high dose of adenosine, they showed no significant difference in stress MBF. In contrast, regadenoson is a selective adenosine A2a receptor agonist, more potent vasodilator than adenosine with a better tolerability profile that has been proposed as a simpler alternative to adenosine infusion to achieve myocardial hyperemia. Previous studies have demonstrated regadenoson non-inferiority to adenosine for the detection of perfusion defects (39, 40) and despite regadenoson selective activity, there is considerable inter-patient variability in hemodynamic response, which seems not to be related to common A2a receptor polymorphisms (41).

**FIGURE 6:**
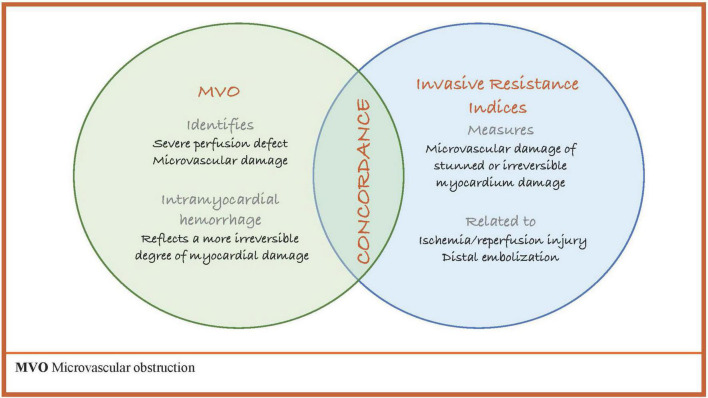
MVO and invasive resistance indices. MVO, microvascular obstruction.

Post-ischemic coronary microvascular dysfunction is a heterogeneous entity that can be found in up to 65% of STEMI patients. A 1% increase in MVO size associates with 14% relative increase in mortality and 8% increase in HF (42), and IMR has been validated to predict MACE following MI (Table 2). However, it is unclear which of either marker of microvascular dysfunction (MVO or invasive resistance indices) or even both should be used to improve risk stratification of MI patients. As previously discussed, invasive physiology often correlates poorly with MVO and a significant proportion of MI cases show discordance from the presence (or absence) of MVO and IMR values (≤40 or above 40). Furthermore, IMR ≤ 40 was associated with IS reduction at 6 months, irrespective of MVO status (14). The wide variability found between invasive and non-invasive markers of microvascular dysfunction suggests that invasive techniques and MVO on CMR measure distinct features of microvascular ischemic pathophysiology, which often occurs concurrently (Figure 6). The recent study by Scarsini (4), further confirmed the impact of MVO or elevated IMR on adverse events after STEMI. Although patients with IMR above 40 and MVO had the worst IS and clinical outcomes at 1 year follow up, patients with either high IMR or MVO, continued to undergo LV remodeling and tended to develop HF after the first year of MI.

Finally, data suggests that coronary hemodynamics are also altered in non-culprit IRA in the acute MI setting (23). These higher IMR values in non-IRA might be explained by MI size, the remodeling of remote myocardium, which occurs in response to increased overload, and by a higher stimulus of neuronal sympathetic axis which promote structural changes in coronary vessels and higher vascular resistances (28). However, abnormal IMR values in the non-culprit vessel seemed not to impact clinical prognosis of MI patients.

## Perspectives

The potential clinical utility of microvascular resistance indices is under continuous investigation. The possibility of having a microcirculation assessment immediately available in the catheterization laboratory, broadens its applicability and interest in the early reperfusion of MI. More research is needed to define the clinical importance of non-IRA microcirculation after an acute MI, and the microcirculation assessment in patients with MINOCA represents an exciting field for future dedicated research.

## Limitations

Since all studies included in this review were observational, non-randomized, with small sample sizes, the readership should anticipate a high risk of type II error. As the lower statistical power of the studies may impede the recognition of microvascular function importance in the MI setting. Regarding the amount of invasive microvascular data available, IMR was disproportionately the most published vascular resistance index. Since HMR and Pzf have no validated cut-off values, different thresholds were used in the clinical trials through ROC curves analysis. The wide range of cut-off values used and the different microcirculation assessment techniques limit the comparison between invasive resistance indices. Moreover, the correlation between CMR findings and hemodynamic markers varied widely (Figure 5), probably due to methodological disparities between studies, raising doubts about the optimal timing for microvascular function assessment and how future clinical trials should be designed. Overall, studies did not consistently report mean ± SD values of microcirculation measurements, which limited the sample size and the performance of meta-analysis for other outcomes in this review. Regarding treatment management, despite most studies reported a similar frequency in the use of standard antithrombotic drugs in primary PCI (aspirin and P_2_Y_12_ inhibitors), the rates of GP IIb/IIIa and aspiration thrombectomy varied significantly between studies (22–71%). Other therapies that potentially impact coronary microcirculation (43) were not consistently reported in our cohort. Moreover, microcirculation studies evaluating clinical outcomes after MI showed a significant heterogeneity in the frequency of use of ACEi/ARA (81–99%), statins (65–94%), and betablockers (74–85%), which unenabled matching patients to their medications.

## Conclusion

Microvascular resistance indices are valuable means to evaluate microcirculation function following MI. Microvascular dysfunction relates to the extent of myocardial damage and clinical outcomes after MI.

## Data availability statement

The original contributions presented in this study are included in the article/Supplementary material, further inquiries can be directed to the corresponding author.

## Author contributions

LP and MS conceived and designed the analysis, data collection, and wrote the manuscript. RT performed and reviewed the analysis and critical revision. MF and LG revised the manuscript critically. All authors contributed to the article and approved the submitted version.
