# Supplementary material for: Microcirculation function assessment in acute myocardial infarction: A systematic review of microcirculatory resistance indices
Source: Front Cardiovasc Med. 2022 Nov 11;9:1041444. doi: 10.3389/fcvm.2022.1041444 (PMC9691675; doi:10.3389/fcvm.2022.1041444)
Supplement: Supplementary file 1 [file Data_Sheet_1.pdf]

Supplementary Table 1 Search strategy

#	Searches using PubMed, Embase and Cochrane Controlled Register of Trials (CENTRAL) databases
**1**	"IMR" OR "Index of microcirculatory resistance" OR "Index of microvascular resistance" OR "Microvascular resistance index" OR "HMR" OR "Hyperemic microvascular resistance" OR "Pzf" OR "Zero-flow pressure"
**2**	"STEMI" OR "ST-elevation myocardial infarction" OR "Acute coronary syndrome" OR "Myocardial infarction"
**3**	#1 AND #2

Supplementary Table 2 Newcastle-Ottawa Scale summary

First author/ study	Selection	Comparability	Outcome
Williams et al. (1)	**	-	**
Patel et al. / OXAMI study (2)	**	-	**
Scarsini et al. (4)	***	-	***
De Maria et al. (10)	**	-	***
McAlindon et al. / MICRO-AMI study (11)	**	-	***
Teunissen et al. (12)	***	*	***
Yoo et al. (13)	**	-	***
Ahn et al. (14)	**	-	***
Fukunaga et al. (15)	**	-	***
Kitabata et al. (16)	**	-	**
Maznyczka et al. (17)	**	-	***
Fearon et al. (18)	**	-	***
Carrick et al. (19)	**	-	***
De Waard et al. (20)	***	*	***
Jin et al. (21)	**	-	***
Ntalianis et al. (22)	**	-	**
Choi et al. (23)	***	*	***
Mejía-Rentería et al. (24)	***	*	***
Díez-Delhoyo et al./ FISIOIAM study (25)	**	-	***
Van der Hoeven et al. (28)	**	-	***
Bax et al. (29)	**	-	***
Scarsini et al. / OXAMI study (45)	**	-	***
Scarsini et al. (46)	**	-	**
Maznyczka et al. (47)	**	-	***
Fahrni et al. /Insights OxAMI study (48)	**	-	***
De Maria et al. (49)	**	-	***
